# The Diagnostic Accuracy of Carotid Doppler in Detecting Anechoic Thrombus Against CT Angiography as the Gold Standard

**DOI:** 10.7759/cureus.26951

**Published:** 2022-07-17

**Authors:** Muhammad Nasir Naeem Khan, Aliya Ahmed, Ibtesam Zafar, Samina Akhtar, Muhammad Haris Aurangzeb, Amir Khan

**Affiliations:** 1 Radiology, Pakistan Institute of Medical Sciences, Islamabad, PAK; 2 Diagnostic Radiology, Pakistan Institute of Medical Sciences, Islamabad, PAK; 3 Cardiology, Pakistan Institute of Medical Sciences, Islamabad, PAK; 4 Radiology, Fedrel Government Polyclinic Hospital, Islamabad, PAK

**Keywords:** cta, anechoic thrombus, carotid artery, doppler, computed tomography

## Abstract

Objective

In this study, we aimed to assess the diagnostic accuracy of carotid Doppler ultrasound (CDU) in detecting anechoic carotid artery thrombus when compared to CT angiography (CTA) as the gold standard.

Materials and methods

This prospective comparative study was conducted at the Radiology Department of the Pakistan Institute of Medical Sciences, Islamabad from January 2022 to May 2022. The study enrolled 32 patients who met the inclusion criteria. We evaluated patients admitted to the neurology ward/OPD who were referred to radiology as part of a stroke workup based on their clinical examination and medical history. In all patients, CDU was used to detect free-floating thrombus (FFT)/anechoic thrombus. CTA was used as the gold standard to assess the diagnostic accuracy of CDU.

Results

The mean age of the study participants was 45.63 ± 7.05 years (range: 33-59 years). Out of 32 patients, 19 (59.4%) were male and 13 (40.6%) were female. The results of CDU were confirmed by CTA in all patients. The diagnostic accuracy of CDU was 53.12% for detecting FFT. The values for sensitivity (54.55%), specificity (50%), positive predictive value (PPV, 70.59%), and negative predictive value (NPV, 33.33%) were also calculated.

Conclusion

Despite the limited sample size, the study concludes that CDU has a diagnostic accuracy of 53%. CTA still remains the gold standard imaging modality for anechoic thrombus if strong clinical suspicion is present.

## Introduction

Around 20% of the blood in the brain's carotid arteries flows on both sides of the neck [1]. An atherosclerotic thrombus may result from the accumulation of plaques along the part of the carotid artery that splits from the carotid bifurcation. Although plaques are generally asymptomatic, some of them can become obstructive. Another form of atherosclerosis that may cause devastating consequences is the development of unstable or free-floating arterial plaques. The free-floating thrombus (FFT) in the carotid artery can rupture or dislodge, causing acute thrombosis, strokes, and even death [2]. An FFT is described as an elongated thrombus attached to the arterial wall by a circumferential blood flow [2].

In the diagnosis of stroke, it is essential to rule out free-floating thrombi since they can appear as anechoic, hypoechoic, or free-floating deposits/areas [3]. Vulnerable plaques are composed of a large, lipid-rich necrotic core, which is covered by thin, inflammation-filled fibrous caps [4] that are infiltrated by macrophages and T cells [5]. This release of cytokines and proteinases stimulates the breakdown of cap collagen and apoptosis of smooth muscle cells, thereby causing plaque rupture [5]. The "vulnerability index", introduced by Naghavi et al. [6], shows whether patients with certain atherosclerosis risk factors are likely to experience a clinical event due to free-floating thrombi. Plaque inflammation, specifically macrophage infiltration, is, therefore, an important predictor of plaque rupture and embolic events [6].

Several methods are currently available for evaluating an FFT, including carotid Doppler ultrasound (CDU), CT angiography (CTA), magnetic resonance angiography (MRA), and digital subtraction angiography (DSA) [7]. Diagnostic methods are chosen depending on the indication and availability in the hospital. CDU is used as a preliminary diagnostic method since it is inexpensive, non-invasive, and readily available [8]. It is difficult to visualize an FFT directly on grayscale ultrasound due to its anechoic nature; however, a CDU shows an absent Doppler signal in the region of the thrombus, indicating an FFT. CDU tests are partially subjective. The physicians interpret the FFT based on their expertise.

A CTA is recommended if CDU confirms or suggests an FFT. CTA is a minimally invasive, objective method for diagnosing free-floating thrombosis. CTA can be used to correct false positives found in CDU, which can often cause mismatches in FFT assessment [9]. The objective of this research was to investigate the diagnostic accuracy of CDU in detecting anechoic carotid artery thrombus with CTA as the gold standard modality.

## Materials and methods

This prospective study was approved by the Ethical Review Board of the Shaheed Zulfiqar Ali Bhutto Medical University - approval no: F.1-1/2015/ERB/SZABMU/915. All adult participants provided written informed consent to participate in this study from January 2022 to May 2022.

The study included all patients over 18 years of age who presented with complaints of sudden numbness of the face, weakness in limbs, dizziness, and headache with the blurring of vision. These patients were sent to the Radiology Department to screen for carotid artery disease through CDU and CTA. We excluded all cases of complete thrombosis, small intraplaque thrombi, smooth mural thrombi, perioperative thrombi, or insufficiently described thrombi.

Using the WHO sample size calculator, the sample size was determined to be 799, which was calculated with a 99% confidence interval, 5% margin of error, and an expected population proportion of 50%. Recruiting so many patients in the short time of the study period proved to be impossible. Hence, all patients presenting to the radiology department with the suspicion of carotid thrombosis were enrolled in the study. We recruited 450 patients for the study through the non-probability sampling technique.

Patients were either admitted to the neurology ward or treated in the outpatient department based on their clinical examinations and medical histories. The Doppler tests were performed in both supine and lateral positions with TOSHIBA® APLIO 500 (Canon Medical Systems Corporation, Ōtawara, Japan). Specifically, 2.5-MHz curvilinear and 5-MHz linear probes were used. An Optima CT540 (GE Healthcare, Chicago, IL) was used for the CTA, with 16 slices scanned. Patients were scanned in the supine position. The contrast was injected with the help of a power injector to avoid any mishaps with the administration.

A total of 450 patients were referred to the Radiology Department during the study period of five months. CDU was used to screen all 450 patients for carotid artery diseases as part of a stroke workup. We found echogenic calcified plaques in 194 patients and echogenic non-calcified smooth plaques in 173 patients. In 61 patients, CDU was found to be normal. We found FFTs in 22 patients only.

Further, 10 patients out of 61 who had normal CDU were advised to be evaluated with CTA by the primary physician as they had strong clinical suspicion of having carotid thrombus based on their significant history of episodes of stroke (four patients) and transient ischemic attacks (six patients). These 10 patients were screened for anechoic thrombus through CTA on the basis of strong clinical suspicion despite having normal CDU.

**Figure 1:**
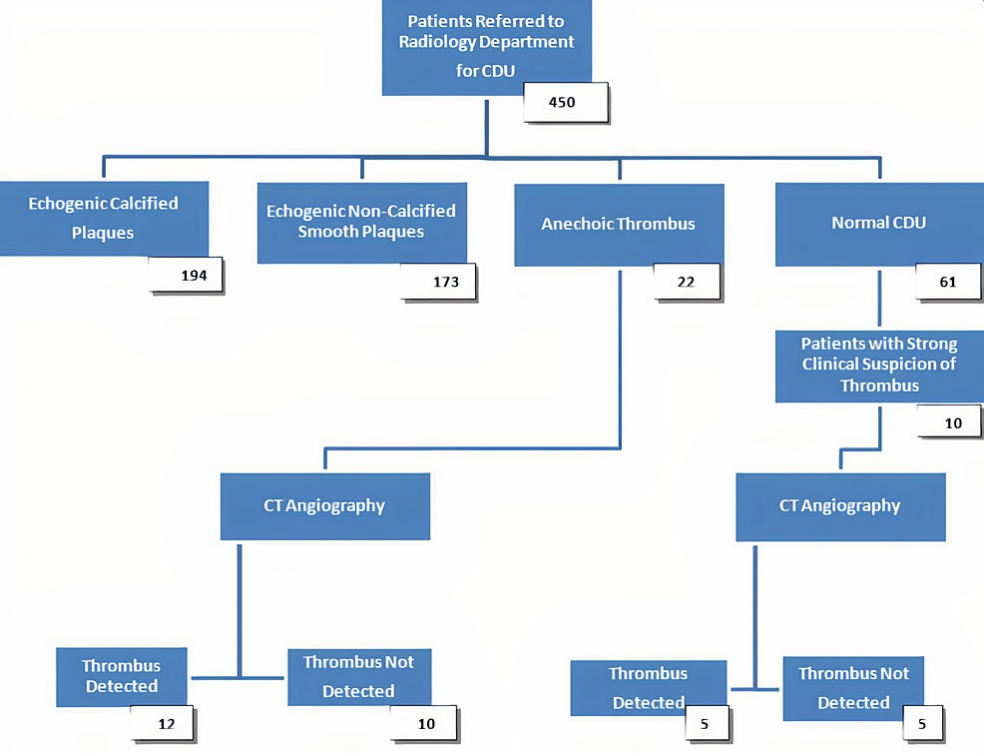
Flow chart for patient segregation CDU: carotid Doppler ultrasound; CT: computed tomography

An Optima CT540 was used for the CTA, with 16 slices scanned. All the scans were completed through IV administration of 100 ml of Iohexol (Omnipaque Injection 350 mg I/ml) with a NEMOTO jet injector (Nemoto Kyorindo co., Ltd., Bunkyo, Japan) at a stream rate of 4 ml/sec. The angiographic scan was performed with the arterial phase commencing almost 15-40 seconds after the administration of the contrast agent. The cylinder current was 180 mA/sec and a cylinder voltage of 120 kV was employed for all patients undergoing CT assessment. The details of patient screening are provided in the chart in Figure 1.

The results of CDU and CTA were compared to determine the diagnostic accuracy of CDU. Data analysis was done using SPSS Statistics version 25 (IBM, Armonk, NY). Mean ± standard deviation (SD) was calculated for continuous variables such as age. Frequency and percentage were calculated for categorical variables. A 2*2 table was created for estimating diagnostic accuracy.

## Results

A total of 32 patients were recruited for comparison between CDU and CTA. The mean age of patients was 45.63 ± 7.05 years (range: 33-59 years). Out of 32 patients, 19 (59.4%) were male and 13 (40.6%) were female, as shown in Table 1.

**Table 1:** Baseline demographic characteristics of patients SD: standard deviation

Variables	Subjects (n=32)
Age, years, mean ± SD	45.63 ± 7.05
Male, n (%)	19 (59.4%)
Female, n (%)	13 (40.6%)

**Figure 2:**
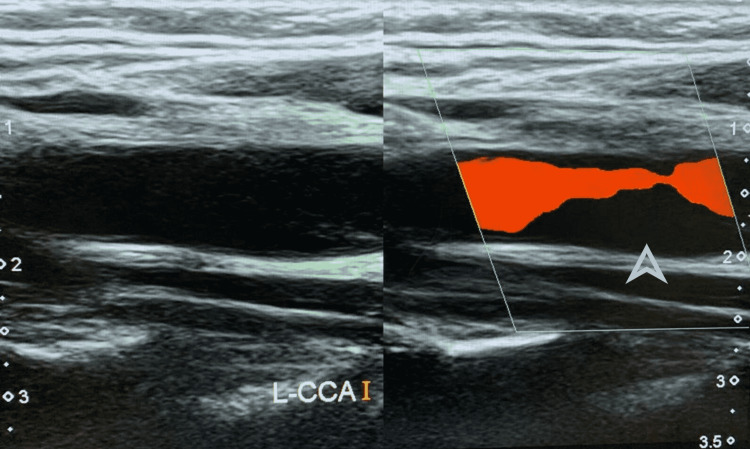
CDU image of a patient showing anechoic thrombus The arrowhead shows an area of no blood flow in the carotid artery on the right-sided CDU image suggestive of a filling defect along the posterior wall CDU: carotid Doppler ultrasound

All patients were examined clinically and their medical history was taken before sending them to undergo CDU. Anechoic thrombus was found in 22 out of these 32 patients by CDU, and 10 patients were negative for thrombus on CDU. Figure 2 shows anechoic thrombus on CDU in a patient. Left-sided image shows no echogenic material in the carotid artery on grayscale. The arrowhead shows the area of no blood flow in the carotid artery on the right-sided CDU image, suggestive of a filling defect along the posterior wall.

**Figure 3:**
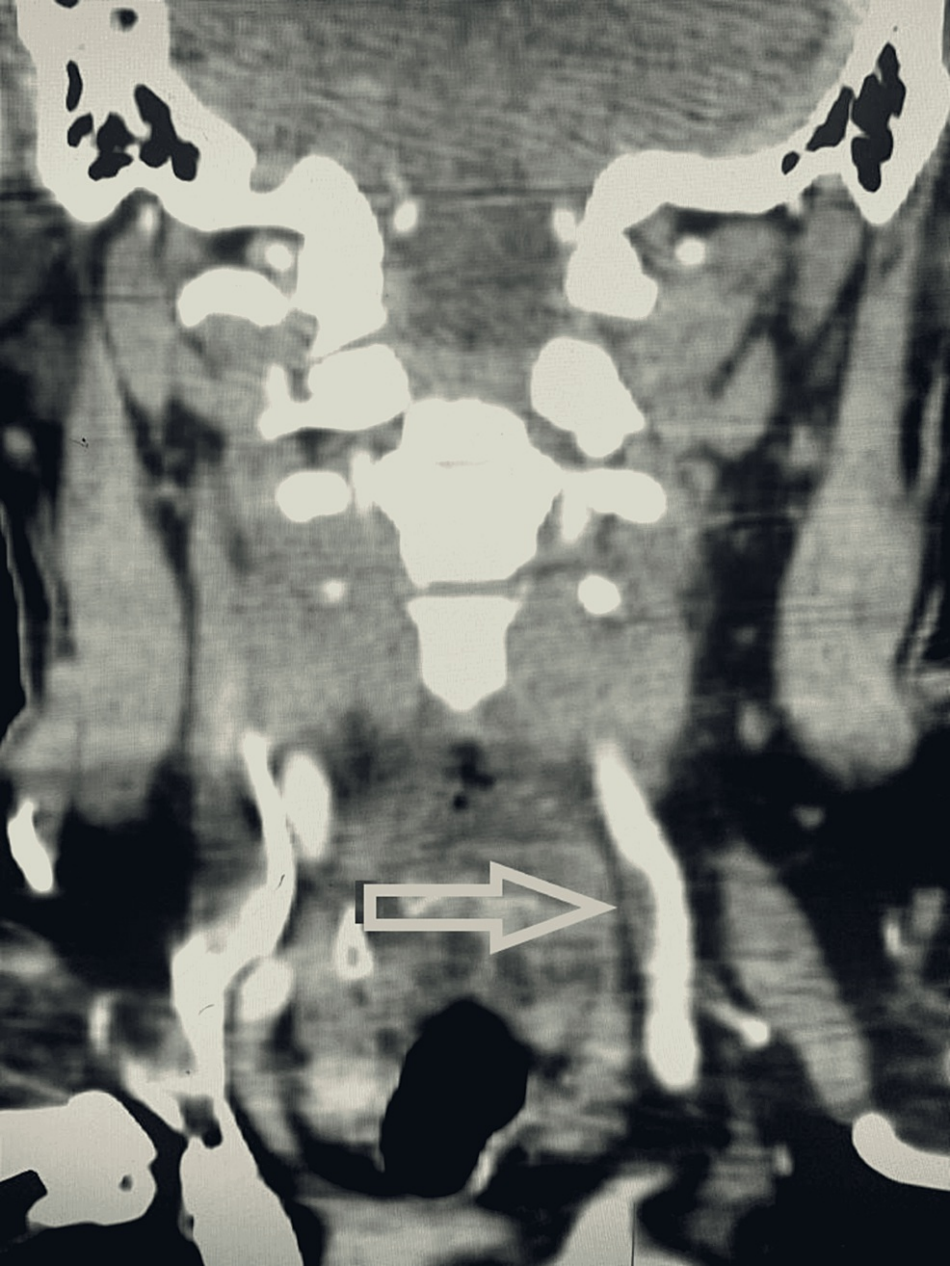
CTA of the same patient shown in Figure 2, confirming the presence of thrombus The arrowhead shows the presence of a filling defect in the left common carotid artery confirming the presence of a thrombus CTA: computed tomography angiography

All patients further underwent CTA to confirm the CDU findings. Out of the 22 patients in whom anechoic thrombus was detected on CDU, 12 patients showed thrombus on CTA, and no thrombus was found in 10 patients. Out of the 10 patients who were negative for thrombus on CDU but underwent CTA, thrombus was detected in five patients on CTA and no thrombus was found in the rest of the five patients. Figure 3 shows the CTA findings of the same patient shown in Figure 2, confirming the presence of thrombus.

Diagnostic accuracy was calculated through a 2*2 table. The values for sensitivity (54.55%), specificity (50%), positive predictive value (PPV, 70.59%), and negative predictive value (NPV, 33.33%) were also calculated, as shown in Table 2.

**Table 2:** Sensitivity, specificity, PPV, NPV, and diagnostic accuracy of CDU against CTA as the gold standard PPV: positive predictive value; NPV: negative predictive value; CDU: carotid Doppler ultrasound; CTA: computed tomography angiography

	Thrombosis detected on CTA	Thrombosis not detected on CTA
Thrombosis detected on Doppler ultrasound	12 (TP)	10 (FN)
Thrombosis not detected on Doppler ultrasound	5 (FP)	5 (TN)
Sensitivity	54.55%	
Specificity	50%	
PPV	70.59%	
NPV	33.33%	
Diagnostic accuracy	53.12%	

## Discussion

Recent advancements in technology, along with improved experience among physicians, have significantly improved the accuracy of CDU for detecting FFT and assessing the risk of cerebral embolism [8]. CDU results that are false-positive are mostly associated with venous thrombosis, as reported in the literature [9]. Usually, atherosclerotic carotid disease affects older adults, and most of the patients we studied presented with transient neurologic symptoms and were of the male gender. Mathiesen et al.'s study also found that males experience carotid FFT more often than females [10]. Cardiovascular history (hypertension, ischemic heart disease, etc.) was the leading risk factor for FFT, followed by stroke and smoking. A recent study suggests that patients with carotid thrombus may be more likely to have cardiac diseases [6]. Bhatti et al. [2] concluded that advanced age, male sex, high blood pressure, hyperlipidemia, and smoking all contribute to FFT; all the patients in our study presented with neurologic symptoms. Other studies have pointed to the history of cardiac diseases as a risk factor for FFT and stroke, which is in line with our study [11].

According to Lane et al., the prevalence of conventional angiography in the diagnostic phase of FFT is high, which reflects the historic bias toward obtaining carotid angiograms [12]. The fact that multiple modalities were used and resulted in concordant findings indicates that angiography is not required and that CTA, MRA, and Doppler imaging are sufficient [13]. Despite a case report in which a stroke was caused by an FFT and Doppler imaging missed it, Doppler remains a safe and non-invasive method [14]. CDU is also a reliable method for diagnosing FFT and its severity; however, CTA is usually recommended to settle all doubts as a reliable method of assessing FFT [15]. The FFT resolved completely in 86% of patients treated medically without any further neurologic progression, according to the literature [16]. Heparin treatment was followed by several weeks to months of warfarin anticoagulation therapy. However, no randomized trials have supported the use of aspirin or antiplatelet agents with this regimen [16,17]. There is no consensus among stroke experts as to whether patients with FFT should be treated with single antiplatelet, dual antiplatelet, or both antiplatelet and anticoagulant therapies in the acute phase. It is also unknown how long any anticoagulant or combined treatment will last [18,19].

Anechoic thrombus is considered to be an unstable fresh thrombus with an increased risk of dislodging and embolism as these are acute thrombi with no fibrous tissue [20]. Our study highlights the importance of timely imaging and workup in cases of anechoic thrombus for appropriate management. The results of the studies mentioned above have been replicated and verified, thereby concluding that CTA and CDU are both useful in identifying FFTs in the carotid artery. This study has a few limitations. It had a small sample size, and a larger number of participants would have led to more conclusive findings. The color Doppler procedure was performed by several examiners, which may have increased the level of subjectivity. Additionally, our data regarding hypercoagulability were limited, although it plays a significant role in FFT pathology.

## Conclusions

Despite its limited sample size, this study concludes that CDU has a diagnostic accuracy of 53%. CTA still remains the gold standard imaging modality for anechoic thrombus if strong clinical suspicion is present.
